# Improving the care of people affected by post-COVID syndrome (LCovB):study protocol of a mixed-methods study

**DOI:** 10.1371/journal.pone.0353270

**Published:** 2026-07-09

**Authors:** Jennifer Marie Burchardi, Martin Brünger, Helma Freitag, Anna Brock, Carmen Scheibenbogen, Natalie Kohzer, Ellen Kexel, Raphael Kohl, Lea Rückgauer, Eva Schmitz, Andrea Budnick, Johanna Schuster

**Affiliations:** 1 Institute of Medical Sociology and Rehabilitation Science, Charité – University Medical Center Berlin, corporate member of Freie Universität Berlin and Humboldt-Universität zu Berlin, Berlin, Germany; 2 Institute of Medical Immunology, Charité – University Medical Center Berlin, corporate member of Freie Universität Berlin and Humboldt-Universität zu Berlin, Berlin, Germany; 3 Department of health care management, BKK Dachverband e.V., Berlin, Germany; Mayo Clinic College of Medicine and Science, UNITED STATES OF AMERICA

## Abstract

**Background:**

The COVID-19 pandemic has resulted in a large number of individuals affected by post-COVID syndrome (PCS) and poses significant challenges to healthcare provision. Many PCS patients are left to manage their condition without adequate support. The aim of the LCovB study is to collect data on symptoms (including pre-existing conditions and comorbidities) and the care situation and trajectories of PCS patients. The results will serve to identify key measures for optimizing care.

**Methods:**

Using a mixed-methods approach, the study includes secondary data analyses of routine data of PCS patients from 44 German statutory health insurance companies, a primary survey and semi-structured interviews with selected PCS patients. In addition, the situation of severely ill patients will be assessed through a physician’s visit and questionnaires. Finally, an expert panel with stakeholders from the healthcare sector will examine the study results using the Delphi method to derive recommendations for treatment and optimized care pathways.

**Discussion:**

The LCovB study examines care trajectories to identify gaps and barriers for patients with PCS, aiming to develop evidence-based recommendations and guidelines for care and disease management, supported by an expert panel.

**Trial registration:**

In August 2025, the study was prospectively registered in the German Clinical Trials Register: Module 1 (DRKS00037341), Module 2 (DRKS00037343), Module 3 (DRKS00037344), Module 4 (DRKS00037345).

## Introduction

According to the Robert Koch Institute (RKI), around 39 million people in Germany have been infected with SARS-CoV-2 since the start of the COVID-19 pandemic in March 2020, of which around 188,000 have died (as at 24 May 2025). According to the RKI, the majority of people with a confirmed SARS-CoV-2 infection are considered to have recovered [1]. However, in the first year of the pandemic, some of those infected already showed persistent symptoms of acute infection, or symptoms that appeared as a result of the infection. The symptoms, subsumed under various terms (including long-COVID), were included by the World Health Organization (WHO) in 2021 under post-COVID syndrome (PCS) (U09.9!) in the International Statistical Classification of Diseases and Related Health Problems, 10th Revision (ICD-10). U09.9! stands for “post-COVID-19 condition” for all cases in which another illness/condition is associated with a previous COVID-19 infection. The diagnosis can be assigned if, three months after a presumed or confirmed SARS-CoV-2 infection, symptoms persist or reappear that last at least two months, cannot be explained by another illness and have a relevant impact on the patient's everyday life [2]. Almost all organ systems can be affected; however, according to the S1 guideline, the most common symptoms include fatigue, cognitive impairment, exercise intolerance, dyspnoea, headaches, disorders of the sense of smell and taste, sleep disorders and psychiatric disorders [2]. The pathophysiological mechanisms of post-COVID symptoms are very diverse and have not yet been finally clarified. Studies have discussed various causes, such as immune dysregulation, mitochondrial dysfunction, prothrombotic inflammation and viral persistence [3].

Previous studies on the prevalence of PCS have produced varying results. The Central Institute for Statutory Health Insurance Physician Care in Germany (Zi) stated a prevalence of around 9% for post-COVID (U09.9!) among all COVID-19 cases registered by 2021 in outpatient claims data in Germany [4]. This number probably reflects underreporting in 2021; studies from European countries such as France, Sweden, Spain, Switzerland, Faroe Islands, Italy and Germany, as well as China and the United States of America came up with prevalence rates between 7 and 30% [5–8]. Hospitalized and female patients are at higher risk of developing PCS [9]. An in-depth analysis by the Zi of outpatient care for PCS patients in Germany, based on claims data from the second quarter of 2021, showed that the majority of people affected are aged 45–64. Persons who were treated for previous somatic and mental illnesses in 2020 also had a higher risk of developing a PCS [10].

In addition, PCS patients were mostly treated exclusively by general practitioners (GPs) (76%). GPs are the first point of contact for those affected, yet studies suggest that they sometimes cannot adequately address the complex problems of people affected by PCS and that there are still too few specialized clinics for the referral of post-COVID cases [11–14]. An increased utilization of pulmonologists and cardiologists in the outpatient claims data compared to a control group was also observed [15].

In 2022, PCS patients most frequently underwent a pneumological rehabilitation, with almost half of the rehabilitations being extended beyond the regular duration [16]. Various studies have shown a significant improvement in post-COVID symptoms through rehabilitation services after a severe course of COVID-19 disease [15,17,18]. The common symptom of fatigue can be reduced in a subset through psychosomatic rehabilitation, but a significant proportion of patients still suffer from relevant fatigue on discharge [19]. A problem in rehabilitation is the lack of knowledge about how to handle post-COVID in those patients who had mild infections and how to approach the frequent symptom of post-exertional malaise (PEM) and the subgroup with ME/CFS. This leads to patients feeling stigmatized or that they are not taken seriously [20]. Adapted, symptom-oriented rehabilitation that avoids overexertion and teaches strategies such as pacing is crucial for PCS patients with PEM. Multimodal and interdisciplinary rehabilitation approaches are seen as promising [21].

This is particularly important in the context of avoiding long periods of work disability among the working population. Although rather low absolute numbers of sick leaves due to post-COVID were registered in initial studies, PCS patients were then unable to work for an average of more than 100 days; depending on gender (more women), the severity of the COVID-19 infection previously contracted and the age of those employed. PCS patients aged 60−64 had a 70 percent higher risk of developing a disability relating to work in the following year compared to 40–44-year-olds [22]. There also appears to be an increased risk of developing cognitive deficits and dementia symptoms with increasing age after surviving COVID-19 infection [23]. Initial findings suggest that PCS patients are moderately to severely limited in their daily activities [24] and that there are particular risk factors for persistent symptoms for those with care and support needs [25].

In addition, reliable data is needed to determine the actual prevalence of PCS as well as more differentiated data on work disability and insights into the coping strategies of PCS patients and their personal perception of care. There is also little analysis of the care providers’ side of the equation.

It is also important to focus analyses on patients with particularly long periods of work disability and/or who are classified as eligible for a defined level of care. It can be assumed that they continue to require adapted care pathways but they have been the subject of hardly any current studies. It is also important to comprehensively examine old and very old PCS patients, their state of health and care trajectories, as well as their prognoses for being classified as needing long-term care.

### Study aims and research questions

The main objective of the LCovB study is to gain a comprehensive understanding of PCS patients’ care trajectories and, based on the results, to derive recommendations for improved healthcare. First, data on the symptoms and care situation of PCS patients will be collected. Second, an expert panel will then use the results to identify key points for optimizing care of PCS patients. A guideline and recommendations for the care of suspected PCS cases, especially for outpatient care, will be developed as long-term goals. Recommendations on disease management will be generated for affected patients.

The following research questions will be examined:

**Main research questions:**

- How large is the proportion of persons with the diagnosis PCS (U09.9!)?
- What are typical healthcare trajectories of PCS patients?

**Further research questions:**

- What are the characteristics of persons affected by PCS (main diagnoses; previous illnesses/comorbidities; symptoms/disease burden; socio-demographic characteristics)?
- Where is the PCS diagnosis most frequently recorded (e.g., in primary care practices)?
- Which particular features characterize old and very old PCS patients?
- Which conditions lead to particularly long-term work disability and/or need for care in PCS patients?
- What regional differences in the healthcare of PCS patients can be identified?
- What personal view do PCS patients have on their healthcare?
- What is the long-term care situation of PCS patients, and can care deficits be identified?
- What improvement measures/potential for PCS patients can be identified from the collected data?

## Methods

**Fig 1:**
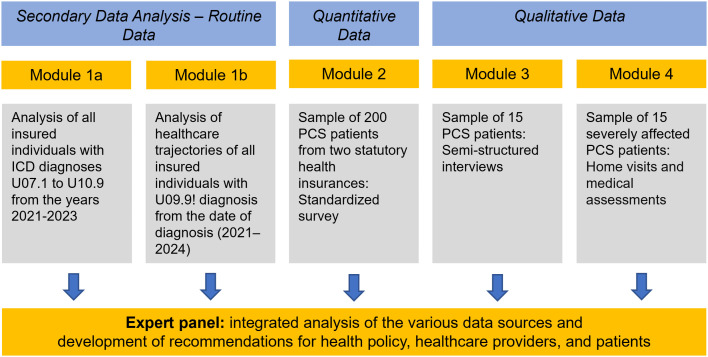
Study design of the LCovB study.

The LCovB study is a mixed-methods study in a parallel design with four sub-studies (Modules) (Fig 1).

### Study design

Routine data analyses of statutory health insurance companies (SHI) in Germany from persons with ICD diagnoses U07.1 to U10.9 (Module 1a) and U09.9! (Module 1b) will be carried out (Fig 1). In Module 2, a quantitative, hybrid primary survey of a sample of PCS patients will be conducted. Module 3 consists of semi-structured interviews with PCS patients and Module 4 will involve medical visits and assessments of a sample of severely ill patients. Finally, recommendations for action, including for the care of the seriously ill, will be derived from the findings of the four Modules by an expert panel, based on a Delphi process. The study will be reported based on the Good Reporting of A Mixed Methods Study (GRAMMS) [26]. The analysis of routine data will be carried out in accordance with good practice for secondary data [27]. The guided interviews will be conducted in accordance with the Consolidated Criteria for Reporting Qualitative Research (COREQ) [28]. The Ethics Committee of the Charité – Universitätsmedizin Berlin, Berlin, Germany (Module 1–3: number: EA1/207/24; Module 4: EA4/166/24) has approved the study protocol. No legal or ethical concerns were identified. The data protection policy was approved after explicit verification and consent by the relevant supervisory agencies. The routine data are transferred in anonymized form and in accordance with Section 75 of the German Social Code Book X (SGB X, § 75 SGB X) after approval of the responsible authorities. All participants of Modules 2, 3 and 4 will receive written information about the study objectives, participation, and their right to decline participation and informed consent will be obtained.

### Participants

The following section describes the inclusion criteria of the different data collections.

#### Module 1 (routine data).

The system of company health insurance funds (“BKK”) is a large association of statutory health insurance companies (SHI) in Germany. The 44 participating SHIs represent 7.8 million insured persons and are all members of the BKK Federal Association (BKK Dachverband e.V.). The socio-demographic data of BKK insured persons show that they do not differ significantly in their characteristics or structure from the respective reference population of all SHI insured persons in Germany. In the BKK insured population, the genders are almost equally represented. The average age is 43.0 years. Half of the BKK insured individuals (49,6%) are employed; 22.7% are family members; 19.8% are retired. In terms of age, gender, and occupational characteristics, employed BKK members do not differ significantly from all employees subject to social insurance contributions in Germany. The only exception is that a higher proportion of BKK members are employed in manufacturing-related occupations, as company health insurance funds are traditionally found primarily in companies in the manufacturing or processing industries. The regional distribution of employed BKK members largely corresponds to that of all employees subject to social insurance contributions in Germany; they are more commonly found in economically strong federal states such as Bavaria [29].

Routine data will be examined of persons who were continuously insured with one of the SHIs participating in the LCovB project from 01.01.2021 to 31.12.2023 (Module 1b: 01.01.2021 to 31.12.2024). In addition, the insured persons must be at least 18 years old at the beginning of the quarter in which the initial diagnosis (outpatient or inpatient) was documented. The two Modules follow different research objectives. Module 1a aims to examine the prevalence of PCS in relation to other diagnoses. Therefore, in the first data delivery for Module 1a, all insured individuals with ICD codes U07.1, U07.2, U08.9, U09.9!, and U10.9 are included. Module 1b focuses on the care trajectories of patients with PCS. Therefore, in the second data delivery for Module 1b, the diagnosis U09.9! serves as the inclusion criterion.

#### Module 2 (survey).

For the standardized survey, a random sample of insured persons of two participating health insurance funds (Pronova BKK and mkk – meine Krankenkasse) are contacted who are aged 18 years or older and for whom the diagnosis U09.9! was documented in the quarter available at the time of the survey with a complete database and at least one other quarter in the previous three quarters (M2Q criterion) [30]. Exclusion criteria are: deceased, not continuously insured during the above-mentioned period, has refused contact by SHI or the use of data for research purposes, lives abroad, has a protection mark (e.g., employee).

#### Module 3 (interviews).

No specific inclusion and exclusion criteria are defined in addition to the presence of a PCS diagnosis and age of 18 years or older. When selecting the interview partners, it is intended that the sample contains different genders, age groups, degrees of severity with regard to the existing PCS symptoms and different durations of PCS.

#### Module 4 (medical visits and assessments).

Patients are selected who meet the criteria for a PCS, were previously healthy, are housebound as a result of the disease (Bell 0–20) and are therefore unable to attend the outpatient clinic, are living in Berlin and wish to participate in the study.

### Data collection

#### Module 1 (routine data).

For the routine data analysis, a complete survey of the insured persons with the corresponding ICD diagnoses at the participating SHI is sought. We use socio-demographic data (§ 288 Social Code Book V) and claims data for healthcare, rehabilitation and nursing services (Social Code Book V and XI) (Table 1).

**Table 1:** Variables of the routine data collection.

Module 1a	Module 1b
• Basic demographic data (year of birth, sex, municipality key, employment status, care level) • Periods of incapacity to work, diagnosis • Outpatient treatment (diagnoses) • Outpatient hospital treatment (diagnoses) • Inpatient hospital treatment (diagnoses)	• Basic demographic data (year of birth, sex, municipality key, employment status, care level) • Periods of incapacity to work, diagnosis • Long-term care claims data • Outpatient treatment, diagnoses and costs • Medication • Outpatient hospital treatment, diagnoses and cost • Inpatient hospital treatment, diagnoses and costs • Rehabilitation (diagnosis, department, start and end of rehabilitation, assessment of work ability) • Therapeutic and assistive aids • Billing data for digital health applications

#### Module 2 (survey).

The hybrid survey is conducted both analogously (questionnaire sent back by post) and, alternatively, online.

The questionnaire surveys sociodemographic factors (e.g., age, gender, family status, migration history, place of residence) and socio-economic factors (educational and vocational qualifications, occupational status, household income). In addition, standardized scales are used, e.g., to measure subjective working capacity and health status, quality of life (QoL), cognitive status, level of activity, pre-existing conditions and accompanying symptoms (Table 2). Some project-specific questions, for example on treatment and satisfaction with healthcare, complement the standardized tools.

**Table 2:** Standardized instruments for the assessment of PCS patients.

Outcome	Instrument
General life satisfaction	Short scale for assessing general life satisfaction (L-1) [31]
Subjective work ability	Work Ability Score (WAS) [32]
Health-related quality of life	Short Form 12 Health Survey (SF-12) [33,34]
Functional capacity	Functional Capacity Questionnaire (FUNCAP27) [35]
Health problems	Self-Administered Comorbidity Questionnaire (SCQ_D) [36]
Psychological well-being	Patient Health Questionnaire (PHQ-4) [37]
Post-COVID-related complaints	Post-Covid Syndrome Score (PCS Score) [38]
ME/CFS diagnosis	SEID criteria [39]
Fatigue	Chalder Fatigue Scale [40]

#### Module 3 (interviews).

The semi-structured interviews are conducted by a researcher via video call or by phone (duration approximately 60 minutes). The semi-structured interview guide is based on the following categories: description of the illness and effects on everyday life (professional, social), perception of healthcare, barriers to using the healthcare system, acceptance and stigmatization, coping with the illness and expectations. The interviews are audio-recorded and transcribed according to Dresing and Pehl [41].

#### Module 4 (medical visits and assessments).

For severely affected patients, a diagnostic workup and an assessment of the nature and severity of the illness are to be conducted via home visits by a physician experienced in PCS and ME/CFS. In preparation for this appointment, patients will complete various digital questionnaires (structured medical history, Bell Scale, FUNCAP55, SF-36, MBSQ/Canadian Consensus Criteria, PHQ-4 Depression, EQ-5D-3L Quality of Life, COMPASS-31, Chalder Fatigue Questionnaire, ESS-Sleep). Patients may receive assistance from relatives with completing the questionnaires and with the organizational preparation of the appointment. Furthermore, the study physician will contact the patient's primary care physician (PCP) and review all prior medical reports.

The clinical evaluation during the home visit includes a comprehensive medical history and a physical examination, which essentially corresponds to the procedure of the ME/CFS diagnostic consultation. The examinations, which include handgrip strength measurement, may be reduced or omitted depending on the patient's level of tolerance, in order to avoid post-exertional malaise (PEM) wherever possible.

Following the appointment, a report will be generated and an individualized treatment plan developed for implementation by the PCP. Over a period of 6 months, the PCP, as well as the patients and their relatives, will receive professional support from the study team for the implementation of this plan. During the same period, the patient's condition will be monitored monthly using the FUNCAP55 questionnaire. At the end of the observation period, a one-time, more comprehensive digital assessment will be performed (Bell Scale, FUNCAP55, SF-36, MBSQ/Canadian Consensus Criteria, PHQ‑4 Depression/Anxiety, EQ-5D-3L Quality of Life, COMPASS-31, Chalder Fatigue Questionnaire, ESS-Sleep).

### Expert panel

The project is supported by an advisory board consisting of members of a patient association and representatives from outpatient care and rehabilitation facilities. This board forms the expert panel responsible for translating the findings of the analyses into practical recommendations for improving patient healthcare.

### Data management

Routine data analyses (Module 1) and analyses of survey data (Module 2) will be conducted using anonymised data. Re-identification of insured individuals will not be possible. Access to all datasets (Modules 1–4) will be restricted to members of the research team only.

### Sample size

#### Module 1 (routine data).

Since the planned routine data analysis involves a complete collection of the data of all insured persons of the participating statutory health insurances companies (SHI), no priori calculation of the sample size is carried out here. It is expected that data of around 13,500 PCS patients of 44 SHIs will be included.

#### Module 2 (survey).

A total of 2,854 people is contacted by post through two SHIs (Pronova BKK and mkk – meine Krankenkasse). Target response size is at least 200 affected individuals. This sample size allows for the determination of frequencies with a 95% confidence interval of approximately 4–7 percentage points (depending on the prevalence) around the point estimate. A conservative response rate of at least 7.5% is anticipated to ensure that, even after excluding invalid data and individuals who do not meet the inclusion criteria, at least 200 individuals can still be included in the analyses. A higher response rate is expected.

#### Module 3 (interviews).

A total of approximately 15 guided, problem-centred interviews is conducted with PCS patients in order to gain a detailed understanding of individual care trajectories and subjectively perceived barriers to care. The planned sample size is based on qualitative saturation principles, the expected information power of a purposively selected sample with heterogeneous symptom experiences but a focused research aim, and previous experiences from qualitative research involving post-COVID patients [42,43]. Data collection and analysis will be conducted iteratively, and thematic saturation will be monitored continuously. If relevant new themes continue to emerge, additional interviews may be considered.

#### Module 4 (medical visits and assessments).

A total of 15 PCS patients in the area of Berlin will be medically consulted and examined in their homes. The planned sample size was chosen to capture a relevant spectrum of severe healthcare situations and functional impairments among homebound PCS patients while allowing for in-depth physician-led assessments. Given the exploratory character of this Module, the substantial time and personnel requirements associated with home visits, and the limited physical capacity of severely affected patients, a sample size of approximately 15 participants was considered feasible and appropriate.

### Data analysis

#### Module 1 (routine data).

First, descriptive statistics will be conducted to describe the sample in terms of sociodemographics and comorbidities. In addition, subgroup analyses are planned. These will include group comparisons regarding symptom severity, disease progression and consequences (e.g., incapacity for work). The groups will be stratified by gender, age group, region (urban versus rural) and among other factors. The focus of all analyses is the PCS diagnosis in combination with other diagnoses, healthcare measures received, and periods of incapacity for work. The relationship between specific constellations of care pathways and symptom severity will be investigated using bivariate and multivariate methods. Binary logistic regressions (dichotomous dependent variable: incapacity for work yes/no) will be used to calculate the strength of various factors influencing the outcome. Further, a cost calculation of care pathways and identification of particularly cost-intensive care units will be carried out.

#### Module 2 (survey).

To analyse the standardized survey, descriptive statistics will be used to provide an overview of the data, and bivariate and multivariate methods (e.g., correlations, regressions) will be used to identify associations. Group comparisons regarding symptom severity, quality of life or health status will be stratified by gender, age or treatment history.

#### Module 3 (interviews).

The qualitative interviews will be analysed using Kuckartz's qualitative content analysis [44] and the MAXQDA software. This combines deductive, theory-driven perspectives with inductive, material-based approaches, thus enabling a differentiated view of the complex reality of healthcare for people affected by PCS.

#### Module 4 (medical visits and assessments).

A descriptive presentation of the physical examination (severity of the disease) and questionnaire surveys will be carried out by the visiting medical staff and a therapy concept initiated.

### Integration of the study findings

After each Module has been analyzed separately according to its own methodological standards, the results are compared and combined to expand our understanding by the convergent design [45,46]. By comparing multiple data types, the strengths of different methods are utilised. The respective results are combined to provide a more comprehensive and meaningful overall picture. The data from the qualitative part is intended to provide contextual information, in-depth insights and individual perspectives of the patients, whilst the data from the quantitative part of the study is intended to reveal prevalence estimates and care pathways. The analysis will examine which information in the data sources is consistent, complementary or contradictory, and this will be discussed and approved by the expert panel. The results are to be linked and presented by joint displays to integrate quantitative and qualitative data [47].

### Expert Panel

For the final development of (care) recommendations, a procedure based on the Delphi method will be used [19]: 1) Initial one-day expert workshop: presentation of the triangulated results of Modules 1–4 and discussion of these results with the experts, and joint development of initial implications for care from these results. Fifteen experts are expected to participate, including representatives of patients and post-COVID outpatient clinics, representatives from pension insurance and rehabilitation, academia, and medical specialists in the fields of general medicine, pulmonology, neurology, psychosomatics, and psychotherapy. At least three patient representatives will be present, and their contributions will be given the equivalent weighting to those of the other experts. 2) Subsequent structuring of the workshop discussion results, and conception and writing of an initial draft of concrete recommendations for action. 3) Structured online Delphi survey involving approximately 20 experts requesting feedback on the individual draft recommendations for action. Consensus will be achieved if at least 70% of the responses are positive. 4) Revision of the recommendations for action based on the feedback. 5) Second structured online Delphi survey requesting feedback on revised recommendations for action. 6) If changes are required to the recommendations: repeat steps 4) and 5) until a final version is agreed upon. 7) Presentation of the final recommendations for action at the closing event.

### Status of the study

At the time of submission, the study is ongoing. Regarding the routine data analyses, record screening and data extraction from the 44 statutory health insurance companies are expected to be completed by May 2026. For the primary data collection (survey and semi-structured interviews), participant recruitment and data collection are ongoing. Overall data collection for all study Modules will be fully completed by May 2026. Final study results are expected to be available by November 2026 for the review by the expert panel.

## Discussion

The aim of the LCovB study is to identify the specific implications for healthcare and to address the still deficient research and data situation on the post-COVID disease field. This will be realised through a comprehensive mixed-methods analysis integrating multiple data sources. Currently, there are no evidence-based, causal treatment options. These are limited to symptom-oriented therapeutic approaches. PCS patients experience significant barriers to accessing healthcare, often related to insufficient medical support and knowledge, lack of recognition and the stigmatization of the disease [11,48–51], which makes it all the more important to improve care pathways.

Using routine data, the care trajectories of diagnosed PCS patients will be analysed. The broad recruitment base allows for the tracking of different care pathways based on the respective diagnosis. This enables the analysis of deficits, over- and undercare, inappropriate care, as well as the identification of best practices. The project also explicitly incorporates the perspectives of PCS patients and self-help groups. Further, insights will be generated into the specific disease situation and care situation for selected affected groups: the seriously ill, the elderly, and patients who become dependent on care as a result of a post-COVID illness. The analyses will form the basis for the development of recommendations and guidelines for care (particularly for practising physicians) and self-management (for patients) specifically targeted at the aforementioned affected groups. These recommendations, based on the insights of a multidisciplinary expert panel, will contribute to the development of needs-based care pathways that more quickly refer affected individuals to the appropriate setting. The research findings will also contribute to strengthening the evidence for necessary changes so that care structures can be further expanded in line with needs (e.g., adapting care services, reintegration into working life). Furthermore, the project results can contribute to the further development of the existing S1 guideline for post-COVID/long-COVID.

Despite the multi-methodological approach, several limitations must be acknowledged. Due to data protection regulations and practical feasibility constraints, it is not possible to establish an individual-level linkage between the routine data and the primary survey data. For this reason, the routine data and the survey data cannot be cross-validated against one another in terms of sociodemographic and healthcare characteristics. However, to address the generalisability, the analysis will include a detailed assessment of the insured persons’ structure (e.g., age, gender, regional distribution), in order to identify potential selection biases. We will compare and analyse the structure of the samples against the structure of statutory health insurance population, the general population and samples from similar studies of people affected by PCS, based on the collected data. Furthermore, the validity of the routine and survey data depends on the quality of diagnostic coding practices, since routine data is primarily generated for billing purposes. Previous research has indicated a potential discrepancy between patient-reported diagnoses and the official ICD codes recorded in insurance databases [52]. Finally, using billing data introduces a temporal lag, which may limit the real-time assessment of the most recent care developments. In the primary survey, the retrospective approach may also introduce recall bias.

## Abbreviations

BKK: Betriebskrankenkasse (Company-based statutory health insurance)
EQ-5D-3L: European Quality of Life 5 Dimensions 3 Level Version
ESS: Epworth Sleepiness Scale
ICD: International Statistical Classification of Diseases and Related Health Problems
FUNCAP: Functional Capacity Questionnaire
GP: General Practitioner
L-1: General Life Satisfaction Short Scale
MBSQ: Munich Berlin Symptom Questionnaire
ME/CFS: Myalgic Encephalomyelitis/ Chronic Fatigue Syndrome
PCP: Primary Care Physician
PCS: Post-COVID Syndrome
PEM: Post-Exertional Malaise
PHQ: Patient Health Questionnaire
QoL: Quality of Life
RKI: Robert Koch Institute
SCQ_D: Self-Administered Comorbidity Questionnaire Germany
SHI: Statutory Health Insurance company
SF-12: 12-Item Short Form Health Survey
SF-36: 36-Item Short Form Health Survey
WAS: Work Ability Score
WHO: World Health Organization
Zi: Central Institute for Statutory Health Insurance Physician Care in Germany
